# IL-6/JAK/STAT3 Signaling in Breast Cancer Metastasis: Biology and Treatment

**DOI:** 10.3389/fonc.2022.866014

**Published:** 2022-03-15

**Authors:** Sara G. Manore, Daniel L. Doheny, Grace L. Wong, Hui-Wen Lo

**Affiliations:** ^1^ Department of Cancer Biology, Wake Forest University School of Medicine, Winston-Salem, NC, United States; ^2^ Wake Forest Baptist Comprehensive Cancer Center, Wake Forest University School of Medicine, Winston-Salem, NC, United States

**Keywords:** breast cancer, metastasis, interleukin-6, JAKs, STAT3, therapeutics

## Abstract

Breast cancer is the most commonly diagnosed cancer in women. Metastasis is the primary cause of mortality for breast cancer patients. Multiple mechanisms underlie breast cancer metastatic dissemination, including the interleukin-6 (IL-6)-mediated signaling pathway. IL-6 is a pleiotropic cytokine that plays an important role in multiple physiological processes including cell proliferation, immune surveillance, acute inflammation, metabolism, and bone remodeling. IL-6 binds to the IL-6 receptor (IL-6Rα) which subsequently binds to the glycoprotein 130 (gp130) receptor creating a signal transducing hexameric receptor complex. Janus kinases (JAKs) are recruited and activated; activated JAKs, in turn, phosphorylate signal transducer and activator of transcription 3 (STAT3) for activation, leading to gene regulation. Constitutively active IL-6/JAK/STAT3 signaling drives cancer cell proliferation and invasiveness while suppressing apoptosis, and STAT3 enhances IL-6 signaling to promote a vicious inflammatory loop. Aberrant expression of IL-6 occurs in multiple cancer types and is associated with poor clinical prognosis and metastasis. In breast cancer, the IL-6 pathway is frequently activated, which can promote breast cancer metastasis while simultaneously suppressing the anti-tumor immune response. Given these important roles in human cancers, multiple components of the IL-6 pathway are promising targets for cancer therapeutics and are currently being evaluated preclinically and clinically for breast cancer. This review covers the current biological understanding of the IL-6 signaling pathway and its impact on breast cancer metastasis, as well as, therapeutic interventions that target components of the IL-6 pathway including: IL-6, IL-6Rα, gp130 receptor, JAKs, and STAT3.

## Introduction

Breast cancer affects 1 in 8 women in the United States and is the second leading cause of cancer-related deaths in women behind lung cancer (1). Breast cancers are diverse and often identified by molecular subtype via immunohistochemical (IHC) expression of prognostic markers: estrogen receptor (ER), progesterone receptor (PR), and the human epidermal growth factor receptor 2 (HER2). The molecular subtypes of breast cancer range in both receptor expression and prognoses: luminal A (ER and/or PR+/HER2-), luminal B (ER and/or PR+/HER+), HER2-enriched (ER-/PR-/HER2+), and triple negative (ER-/PR-/HER2-) (2). HER2-enriched breast cancer and triple-negative breast cancer (TNBC) are the most aggressive subtypes represented by a higher Ki67 staining, poorer patient survival, and the highest propensity to metastasize (3). Primary breast cancer patients have a 5-year survival rate of 99%; however, the development of metastases diminishes survival rates to 28% (1). 20-30% of breast cancer cases metastasize to distant organs, which accounts for 90% of breast cancer-related deaths (4). The most common sites of metastasis for breast cancer patients include the bone, lung, brain, and liver (5). The development of metastases is a complex process comprised of multiple steps including epithelial-mesenchymal transition (EMT), local invasion, migration, intravasation, extravasation, mesenchymal-epithelial transition, and colonization to a distant organ. During many of these steps, breast cancer cells secrete small soluble proteins, such as cytokines, to promote cancer cells (autocrine effect) and prime microenvironmental cells (paracrine effect) (6). Cytokines are secreted, pleiotropic proteins (15-20 kDa) that mediate a myriad of immunological and inflammatory responses that are often hijacked in cancer. Many cytokines can exhibit either pro- or anti-cancer properties, including interleukin-6 (IL-6). Under homeostatic conditions, IL-6 plays fundamental roles in immune response, inflammation, hematopoiesis, and bone homeostasis; however, dysregulation of IL-6 promotes the pathogenesis of multiple inflammatory and immune-mediated diseases, as well as, cancer (7, 8). The IL-6 signaling pathway is one of the most dysregulated pathways in cancer. For example, IL-6 is elevated in sera of ovarian, cervical, colorectal, esophageal, head-and-neck, pancreatic, prostate, liver, lung, gastric, and breast cancer patients (9–24). In breast cancer, IL-6 expression correlates with poor patient survival, promotes growth and invasion, and mediates metastatic progression, which identifies the IL-6 signaling axis as a potential therapeutic target (25, 26). Consequently, many IL-6-pathway-targeted therapies have been developed and evaluated for breast cancer. Herein, we summarize the biology of the IL-6 signaling pathway, its roles in breast cancer metastasis, and therapeutic advancements in targeting the IL-6/JAK/STAT3 signaling axis.

## IL-6 Signaling

IL-6 was first identified as a 26 kDa T cell-secreted factor that stimulates B cells for antibody production. Since the cloning of IL-6 cDNA by the Hirano and Kishimoto group in 1986 (27, 28), it became evident that IL-6 function was not limited to the immune system as the cDNA sequence maintained homology to other identified proteins: B cell stimulatory factor-2, Hepatocyte-stimulating factor, Hybridoma plasmacytoma growth factor, and interferon β2 (28–32). IL-6 is a member of the IL-6 cytokine family, a four-α helical bundle cytokine, and is secreted by both immune and non-immune cells. The IL-6 cytokine family encompasses eight cytokines, namely, IL-6, IL-11, ciliary neurotrophic factor (CNTF), leukemia inhibitory factor (LIF), oncostatin M (OSM), cardiotrophin 1 (CT-1), cardiotrophin-like cytokine (CLC), and IL-27. These cytokines bind to their respective receptors, but all utilize the signal-transducing co-receptor, glycoprotein 130 (gp130, CD130, IL-6Rβ, IL6ST, 130 kDa). More recently, two additional cytokines were added to the IL-6 cytokine family, IL-35 and IL-39, which utilize gp130 for signal transduction (33, 34). Specifically, IL-6 requires both interleukin-6 receptor α (IL-6Rα, CD80, 80 kDa) and gp130 to activate the downstream pathways *via* classic signaling, trans-signaling, or trans-presentation (35–37).

### Classic Signaling

**Figure 1:**
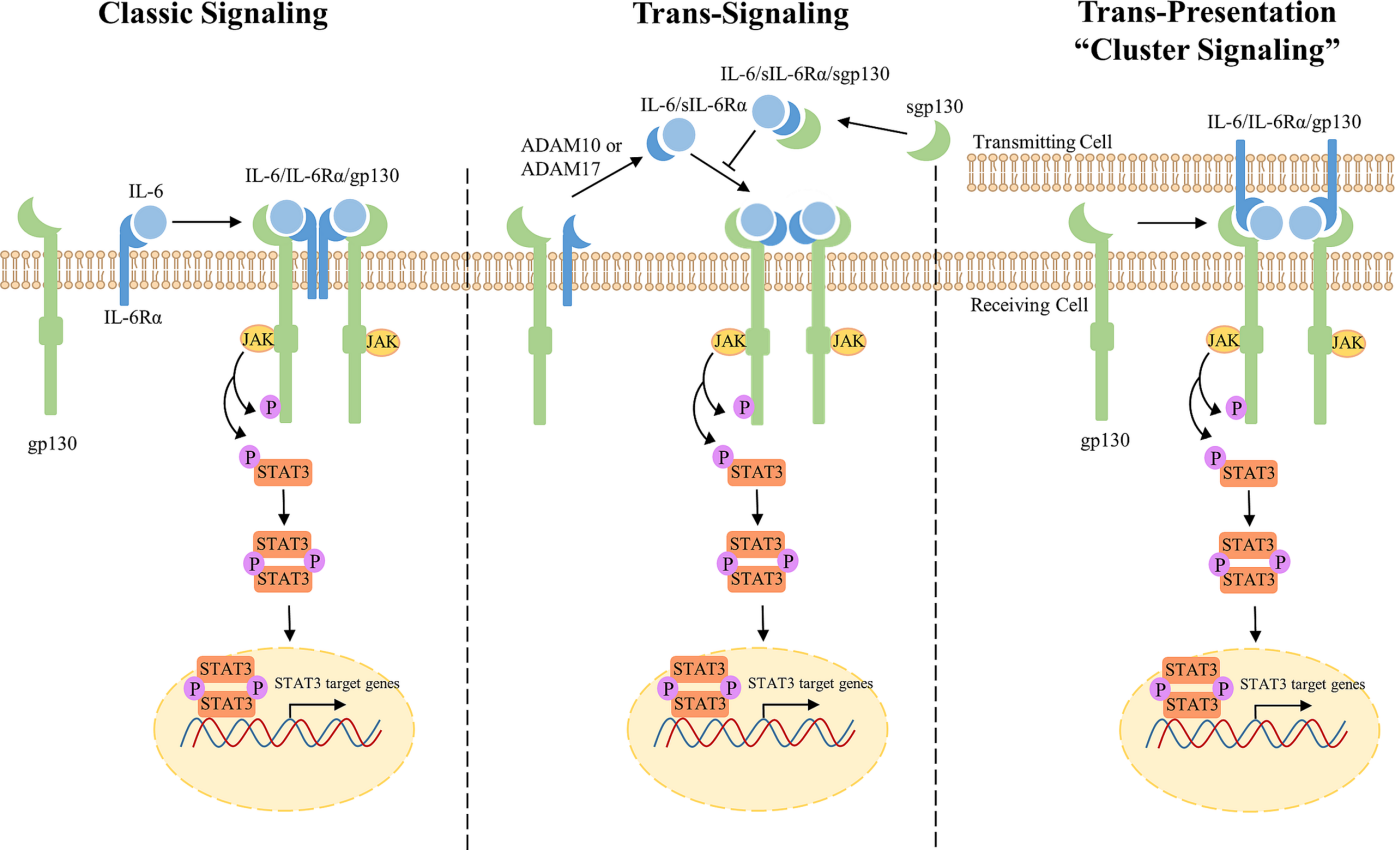
Overview of IL-6/JAK/STAT3 Classic, Trans-signaling, and Trans-presentation. Classic Signaling (left) occurs when IL-6 binds membrane-bound IL-6Rα leading to the subsequent formation of a trimeric receptor complex with signal-transducing subunit, gp130. Two trimeric IL-6/IL-6Rα/gp130 complexes bind through the D1 domain of gp130 to form a hexameric receptor complex for intracellular signaling through the JAK/STAT3 pathway. JAKs are recruited to the membrane and phosphorylate the cytoplasmic tail of gp130 and STAT3. pSTAT3 homodimerizes and translocates into the nucleus for activation of transcription. Trans-signaling (middle) occurs when IL-6Rα presents in a soluble form through mRNA alternative splicing or proteolysis by ADAM10/17. IL-6 binds sIL-6Rα to form a hexameric receptor complex through membrane-bound gp130 for signal transduction. Sgp130 antagonizes IL-6 signaling through sequestration of IL-6/sIL-6Rα. Trans-presentation (right), or “cluster signaling,” occurs between two different cells. A gp130 receptor complex on a receiving cell responds to a IL-6/IL-6Rα complex on a transmitting cell to induce downstream STAT3 signaling.

Classic IL-6 signaling is mediated strictly through membrane-bound receptors, IL-6Rα and gp130 (**Figure 1**, left) (38). IL-6 first binds to IL-6Rα on the cell surface which creates a high affinity for transmembrane gp130. Two trimeric receptor complexes (IL-6/IL-6Rα/gp130) homodimerize; IL-6 of one trimeric complex binds to the D1 domain of gp130 of the second trimeric complex, forming a signal transducing hexameric receptor complex (39). The IL-6/IL-6Rα/gp130 receptor complex activates mitogen activated protein kinase (MAPK), phosphatidylinositide-3-kinase (PI3K), Janus kinases (JAKs), and signal transducer and activator of transcription (STATs) signaling cascades. Formation of the IL-6/IL-6Rα/gp130 hexameric complex recruits the JAK family of non-receptor tyrosine kinases (JAK1, JAK2, and TYK2) to the membrane which associate with and phosphorylate the cytoplasmic tail of gp130 at five tyrosine residues (Y759, Y767, Y814, Y905, and Y915) (40). Phosphorylated gp130 serves as a docking site for STAT1 and STAT3 transcription factors that are subsequently phosphorylated by JAKs at Y701 and Y705, respectively (41, 42). Notably, IL-6 activates STAT3 more potently when compared to STAT1 (43). Upon phosphorylation, STAT3 undergoes a conformational change, detaches from the receptor complex, and homodimerizes allowing for STAT3 translocation into the nucleus to promote transcriptional activation (44). STAT3 is negatively regulated by tyrosine phosphatases, disruption of JAKs and/or cytokine receptors by suppressors of cytokine signaling (SOCS), or direct protein inhibitors of activated STATs (PIAS) (45–47). Receptor availability can also become a limiting factor for IL-6 signaling since IL-6 must be complexed with IL-6Rα in order to bind to gp130 receptor for signal transduction (38). Interestingly, transmembrane gp130 expression is ubiquitously expressed on most cell types; however, expression of membrane-bound IL-6Rα is restricted, therefore, limiting classic signaling to a small subset of cells (48, 49). Since IL-6 modulates pleiotropic effects beyond immune cells, it quickly became evident that IL-6 signals *via* alternative mechanisms outside of membrane-bound receptors, termed trans-signaling.

### Trans-Signaling

IL-6 trans-signaling is mediated through a soluble form of IL-6Rα (sIL-6Rα) to potentiate IL-6 signaling in cells lacking sufficient expression of membrane-bound IL-6Rα (**Figure 1**, middle) (38, 50). Originally detected in serum and urine samples, sIL-6Rα functions as an agonist for IL-6 signaling. sIL-6Rα is produced either by proteolytic cleavage of the membrane-bound IL-6Rα or alternative splicing of pre-mRNA (50–54). Membrane-bound IL-6Rα undergoes proteolysis, or shedding, by disintegrin and metalloproteinase domain-containing proteins ADAM10 or ADAM17 to form sIL-6Rα (50, 55–57). Secreted IL-6 binds to sIL-6Rα which binds transmembrane gp130. Subsequently, two trimeric receptor complexes homodimerize to activate downstream signaling. Interestingly, gp130 can also present in a soluble form (sgp130) and sequesters IL-6/sIL6Rα, thus antagonizing IL-6 trans-signaling without impacting IL-6 classic signaling. However, sgp130 levels are almost negligible when compared to sIL-6Rα (52, 58). Trans-signaling regulates the IL-6 immune response and mediates pro-inflammatory responses through recruitment of mononuclear cells, stimulation of endothelial cells, T-cell survival, and inhibition of regulatory T-cell differentiation (59, 60). Administration of IL-6 and sIL-6Rα activates STAT3 in endothelial cells, solely expressing membrane-bound gp130, to recruit leukocytes for local inflammation *in vitro* and *in vivo* (61). Since trans-signaling mediates the pro-inflammatory responses induced by IL-6, trans-signaling is referred to as the primary mechanism by which IL-6 signaling promotes tumorigenesis in multiple cancers (59, 62, 63). In cancer, IL-6 trans-signaling induces therapeutic resistance, angiogenesis, and is associated with poor clinical outcome (64).

### Cluster Signaling

More recently, a third mechanism of IL-6 signaling has been reported where IL-6 signals between two interacting cells termed, trans-presentation or “cluster signaling” (37). Originally discovered in 2017, Heink et al. discovered IL-6 binds membrane-bound IL-6Rα on one cell (transmitting cell) and is able to bind a gp130 receptor on another cell type (receiving cell) for signal transduction (**Figure 1**, right) (37). Co-culture experiments identified that gp130 receptors on T cells responded to IL-6/IL-6Rα complexes on the membrane of dendritic cells resulting in robust activation of STAT3. Functionally, IL-6 cluster signaling allowed dendritic cells to prime pathogenic T helper 17 cells. Since sgp130 is known to antagonize IL-6 trans-signaling, but not classic signaling, Heink et al. also investigated whether sgp130 is able to neutralize IL-6 cluster signaling. Although sgp130 did not show inhibitory effects in this model, Lamertz et al. reported sgp130 to neutralize IL-6 cluster signaling by directly binding to the IL-6/IL-6Rα complex on a transmitting cell (65). Given these contradictory findings, the IL-6 trans-presentation mechanism in addition to its biological and pathogenic roles remains to be characterized and elucidated.

## IL-6 Signaling in Breast Cancer

Breast cancer is the most commonly diagnosed cancer in women. Despite recent advancements in targeted therapeutics, remission and survival in metastatic breast cancer patients remains poor (1). Metastatic dissemination can be regulated by multiple mechanisms including uncontrolled inflammation in the breast tumor microenvironment through the secretion of chemokines, growth factors, and cytokines to mediate immune evasion and promote tumor progression (66, 67). Importantly, the tumor microenvironment is comprised of a myriad of cell types, such as tumor-associated macrophages (TAMs), helper T cells, bone marrow-derived cells, adipocytes, fibroblasts, and cancer cells which secrete pro-inflammatory cytokines, such as IL-6. IL-6 can be secreted in an autocrine or paracrine manner by both immune and non-immune cells in the tumor microenvironment. The mechanisms by which IL-6 mediates crosstalk between the tumor microenvironment and tumor cells continues to be investigated to develop therapeutic targets in breast cancer. Specifically, IL-6 is of particular interest due to its increased levels in sera of breast cancer patients when compared to normal sera or tissue of healthy patients (68, 69). Increased IL-6 levels can be a result of single nucleotide polymorphisms (SNPs) in the promoter region of IL-6 gene, and have been demonstrated to predict poor prognoses in breast cancer patients. *IL-6* SNPs are significantly associated with ER-positivity and results in a worse disease-free survival (70). Specifically, the rs1800795 SNP in *IL-6* is associated with an increased risk of breast cancer metastasis, irrespective of ER status (71). Furthermore, IL-6 is upregulated in sera of patients with advanced stages of breast cancer and in patients presenting with metastases (68, 72). Notably, patients with metastases at two or more sites have increased IL-6 sera levels compared to patients presenting with one metastasis, and high levels of IL-6 correlates with significantly worse survival in metastatic breast cancer patients (72–74). In addition to IL-6, clinicopathological analyses have been conducted on IL-6’s corresponding receptor, IL-6R. Labovsky et al. identified IL-6 to positively correlate with IL-6R in breast cancer specimens, and that IL-6/IL-6R are co-overexpressed in breast carcinomas when compared to normal mammary tissues (75). Consequently, higher serum levels of sIL-6R predicts a shorter relapse-free survival in ER+ breast cancer patients (76). To complement above findings, stromal expression of IL-6Rα in primary breast carcinomas has been reported to be significantly correlated with metastatic occurrence, and a worse disease-free and overall patient survival (77). Moreover, IL-6 has also been reported to correlate with therapeutic resistance in breast cancer patients highlighting the IL-6/JAK/STAT3 pathway as an important prognostic marker in breast cancer progression, chemoresistance, and metastatic formation (78).

### IL-6 Activation of STAT3

In pathophysiological states, IL-6 mediates inflammation while concurrently regulating MAPK, PI3K, and JAK/STAT oncogenic pathways (79). STAT3 is a primary downstream regulator of IL-6 signaling with its distinct role in regulating inflammation and neoplastic transformation (80, 81). Although STAT3 activation is tightly regulated under homeostatic conditions, overexpression of upstream effectors such as IL-6, IL-6Rα, or gp130 or loss of negative regulators (SOCS, PIAS, etc.) can lead to aberrantly activated STAT3 (82). IL-6 and phosphorylated-STAT3 (phospho-STAT3, pSTAT3) are co-overexpressed in primary breast cancer specimens (83). Upstream regulators that mediate STAT3 activation include canonical cytokines, growth factors, G-protein-coupled receptors, and microRNAs (miRNAs); however, IL-6 remains the primary activator of STAT3 signaling (84). For example, conditioned medium from IL-6-positive breast cancer cells stimulated STAT3 phosphorylation in IL-6-negative breast cancer and non-cancerous epithelial cells, while administration of anti-IL-6 antibodies abrogated these effects (85). Furthermore, homozygous STAT3 knockout tumors presented with decreased tumoral IL-6 expression and reduced systemic IL-6 levels in an orthotopic mammary fat pad (MFP) syngeneic mouse model (86). Functionally, the IL-6/JAK/STAT3 signaling axis promotes proliferation, angiogenesis, EMT, and the cancer stem cell (CSC) subpopulation, while concurrently suppressing the antitumor immune response (87–90). Secreted IL-6 induces expression of STAT3 target genes such as cyclin D1, Bcl-2, Bcl-xL, VEGF, VEGFR2, and matrix metalloproteinases (MMPs) (91–94). STAT3 is aberrantly active in breast cancer and promotes cancer growth through transcriptionally regulating target gene expression resulting in induction of G1 cell cycle progression, proliferation, anti-apoptosis, angiogenesis, and metastasis (95–97). Dysregulated STAT3 activates immunosuppressive tumor-infiltrating myeloid-derived suppressor cells (MDSCs), TAMs, and T regulatory cells; STAT3 further induces expression of upstream cytokines and growth factors creating a vicious autocrine and paracrine positive feedback loop (86, 98–101). In addition to STAT3, IL-6 can be further activated through nuclear factor kappa B (NF-κB) signaling in breast cancer. IL-6 is repressed by the let-7 miRNA, which targets the 3’-untranslated region (UTR) of IL-6 mRNA. Activation of NF-κB represses let-7 and results in super-activation of IL-6 and subsequent activation of STAT3 (102). OSM can also further activate IL-6/JAK/STAT3 signaling both *in vitro* and *in vivo* to promote breast cancer progression. More specifically, OSM synergizes with IL-1β to induce IL-6 secretion in ER+ and TNBC cells for further STAT3 activation (25). Because STAT3 is constitutively active in the majority of breast cancers and plays an important role in mediating breast cancer growth, migration, and metastasis, this review will focus on the IL-6/JAK/STAT3 signaling cascade (95, 103, 104).

### IL-6/JAK/STAT3 Role in Breast Cancer EMT and CSCs

Whether IL-6 enhances or inhibits breast cancer cell proliferation *in vitro* remained controversial for several years. Early studies reported recombinant IL-6 to inhibit or have no significant effect on breast cancer cell proliferation (105–107). More recently, it has become widely accepted IL-6 mediates an oncogenic role in multiple cancers, including breast cancer, primarily through the activation of STAT3. Since IL-6Rα and gp130 are required for signal transduction, the previous contradictory results may have been attributed to a lack of receptor expression in tested breast cancer cell lines, or that IL-6’s pleiotropic effects may depend on JAK/STAT3 pathway activation. For example, gp130 suppresses cell-cycle progression by upregulating G_1_ cyclin/cyclin-dependent kinase (CDK) inhibitor, p21, independent of STAT3. In stark contrast, gp130-induced STAT3 signaling regulates cell cycle transition from G_1_ to S phase by upregulating cyclins D2, D3, A, and cell division cycle 25 A (CDC25A) while simultaneously downregulating CDK inhibitors, p21 and p27, indicating contradictory roles that may be regulated by the balance of STAT3 (108).

STAT3 is involved in proliferation and suppression of apoptosis of breast tumor cells through the upregulation of target genes cyclin D1, c-myc, Mcl-1, Bcl-2 and Bcl-xL (92, 109, 110). STAT3 also upregulates MMP2, MMP9, Twist, Snail, and vimentin expression to mediate an EMT phenotype (87, 95, 111, 112). To complement these findings, STAT3 knockdown resulted in a decrease in CD44+ subpopulation, mammosphere formation, and protein expression of stemness genes Oct-4 and Sox-2 in breast cancer cells (113). Sullivan et al. also reported IL-6 induces the EMT phenotype in breast cancer cells, and found IL-6 overexpressing breast cancer xenografts exhibited decreased E-cadherin and increased vimentin protein expression (114). More recently, Cho et al. utilized a microfluidic chip, which mimics the breast cancer microenvironment and breast metastatic phenotypes *in vitro*, and found that IL-6-treated breast cancer cells successfully invaded into blood and lymph vessels mimicking breast cancer lymphatic metastasis *in vitro* (115). Others have reported IL-6 to promote breast cancer cell proliferation, migration and invasion, and CSCs in breast cancer (116–118). Notably, breast CSCs are known to play a significant role in tumor recurrence and therapeutic resistance (119). Interestingly, mammospheres derived from node-positive breast carcinomas express higher levels of IL-6 when compared to their respective non-malignant matched mammary tissues indicating IL-6 may play a role in CSC renewal (120). Many studies have further validated IL-6 as a key regulator of breast CSCs. For example, IL-6 enriches the breast CSC subpopulation where administration of IL-6 stimulates spheroid growth in MCF-7 cells (120). To complement these findings, IL-6 enriches CD44+ cells as well as an EMT phenotype in breast cancer *in vitro* (114, 121). To determine if IL-6-mediated breast CSCs translates *in vivo* utilizing mouse metastasis models, Korkaya et al. demonstrated PTEN knockdown activates the IL-6 inflammatory loop which, in turn, promotes the breast CSC subpopulation in HER2+/trastuzumab-resistant cells, tumor growth, and secondary metastases *in vivo* (122).

In addition to breast CSCs, surrounding microenvironmental cells are known to communicate with and prime secondary organs for metastatic dissemination. Multiple mechanisms mediate IL-6-induced breast cancer progression, such as activation of autocrine/paracrine loops under inflammatory conditions and IL-6’s impact on the surrounding tumor microenvironment. For example, the oncogene Multiple Copies in T-cell Malignancy-1 (MCT-1), a recently identified prognostic biomarker in aggressive breast cancers, stimulates M2 macrophages in the tumor microenvironment through stimulation of IL-6. IL-6 promotes M2 macrophage polarization while concurrently stimulating TNBC stemness and tumor progression (123). IL-6 also functions in a paracrine manner to promote an invasive phenotype in breast cancer. For example, adipose stromal cells secrete IL-6 to promote breast cancer migration and invasion *in vitro* using the TNBC cell line, MDA-MB-231 (124). Further findings confirmed adipocyte-secreted IL-6 induces EMT in luminal A and TNBC cells through the activation of STAT3 (125). Additionally, isolated fibroblasts from breast tissue and breast cancer metastases secrete significantly more IL-6, enhance breast cancer cell growth, and induce pSTAT3 when compared to normal skin fibroblasts supporting IL-6’s role in priming the “soil” for organ-specific metastasis (126). Taken together, the IL-6/JAK/STAT3 pathway is a major regulator of breast cancer metastasis through promoting breast cancer cell proliferation, EMT, enriching the breast CSCs, and suppressing apoptosis.

### IL-6/JAK/STAT3 in Metastatic Breast Cancer Mouse Models

Given that the IL-6/JAK/STAT3 signaling axis promotes metastatic phenotypes of breast cancer, researchers have investigated the role of IL-6/JAK/STAT3 in breast cancer metastasis *in vivo* to identify key drivers and therapeutic interventions. IL-6 has been demonstrated to prime distant sites for metastatic formation. For example, tumor secreted IL-6 has been recently reported to enhance metastatic potential through educating monocyte-dendritic progenitors to prime distant organs for breast cancer metastasis. Magidey-Klein et al. demonstrates IL-6 plays a functional role in mediating crosstalk between primary tumors and the bone marrow to promote monocyte-dendritic progenitors to give rise to immunosuppressive macrophages which, in turn, promotes metastasis *in vivo* (127). Whether IL-6 mediates organ-specific breast cancer metastasis remains to be conclusively elucidated. However, due to the prominent role of IL-6 in bone metabolism and homeostasis, it is accepted IL-6 is associated with bone metastases and osteoclastogenesis. Dysregulated IL-6 promotes a pro-tumorigenic role in the bone microenvironment allowing breast cancer cells to invade the bone. Consequently, breast tumor cells secrete IL-6 in a paracrine manner to activate osteolytic target genes, namely, PTHrP, RANKL, and DKK-1 (128). In addition to breast cancer bone metastases, recent functional studies have demonstrated IL-6 to promote lung metastases in breast cancer *in vivo*. Siersbæk et al. conducted an intraductal xenograft implantation of an ER+ breast cancer cell line overexpressing IL-6 and found a significant increase in pSTAT3 in the primary tumors, as well as, an increase in metastases in the lung *in vivo* (129). Moreover, another group investigated metastatic potential of IL-6 *via* an intravenous injection of a TNBC cell line overexpressing IL-6, and found tumors to significantly increase lung metastases (86). To complement these findings, Lin et al. reports CGI-99, or C14orf166, enhances IL-6 transcription resulting in hyperactivation of IL-6/STAT3 signaling to promote lung metastases (130). There are multiple downstream effectors under current mechanistic investigation for their role in mediating IL-6-induced metastatic phenotypes in breast cancer. For example, Nyati et al. identified a novel downstream long noncoding RNA, AU021063, which is induced by IL-6 to promote breast cancer metastasis *in vivo* (131). While multiple models are are currently under investigation, mechanistic mouse models expand beyond the scope of this review. The emerging role of IL-6/JAK/STAT3 in promoting the breast CSC subpopulation and breast cancer metastases *in vivo* underscores the therapeutic potential in exploiting the IL-6/JAK/STAT3 signaling axis in metastatic breast cancer.

### IL-6/JAK/STAT3 in Therapeutic Resistance

Since IL-6/JAK/STAT3 signaling upregulates breast CSCs which are known to mediate metastasis and therapeutic resistance, it is to no surprise that IL-6 has been shown to play a role in chemoresistance. IL-6 secretion and expression is significantly elevated in therapeutically resistant breast cancer cells when compared to their respective parental lines. Furthermore, administration of recombinant IL-6 induced chemoresistance through upregulation of drug-resistant gene, *mdr1*, in breast cancer cells (132). Wang et al. identified STAT3 induces breast CSC renewal and chemoresistance through upregulation of fatty acid β-oxidation; administration of leptin resensitized breast tumors to chemotherapy *in vivo* (133). Furthermore, activation of the IL-6 inflammatory loop induces trastuzumab-resistance in HER2+ breast cancer cells indicating that IL-6’s pro-inflammatory role mediates breast cancer therapeutic resistance (122). Given these findings, studies have aimed to utilize IL-6/JAK/STAT3 signaling inhibition in combination with current standard-of-care (SOC) treatment (**Tables 1**, **2**). Administration of Bcl-2 antagonist, sabutoclax, concurrently suppresses IL-6/STAT3 signaling to resensitize chemoresistant breast cancer cells to chemotherapeutic agents (175). Using a resistant ER+ patient-derived xenograft (PDX) mouse model, Siersbæk et al. reported a significant reduction in tumor growth with treatment of STAT3 inhibitor, ruxolitinib, but not fulvestrant alone, a SOC treatment for ER+ breast cancer (129). Clinically, cytoplasmic staining of IL-6Rα is significantly correlated with tamoxifen resistance in ER+ breast cancer patients suggesting IL-6/JAK/STAT3 is an actionable therapeutic target to sensitize tumor cells to current SOC treatment (78). To summarize, the IL-6/JAK/STAT3 signaling pathway mediates breast cancer progression, metastasis, and therapeutic resistance thus justifying investigations into IL-6/JAK/STAT3 as a targeted therapy for breast cancer patients.

**Table 1:** Targeting IL-6/JAK/STAT3 Signaling in Preclinical Breast Cancer Models.

Compound	Target	Models Used	Citation
Siltuximab	IL-6	Human marrow stromal-cell conditioned MCF-7 engraftment in MFP xenograft mouse model as single agent and in combination with Fulvestrant; Treatment in six orthotopically implanted PDX lines *in vivo*.	(134, 135)
MEDI5117	IL-6	Treatment as a single agent in MCF-7 xenograft, combination with taxanes or gefitinib in KPL-4 orthotopic mouse model, and trastuzumab-resistant breast tumor xenograft mouse model (BT474-PTEN-LTT).	(136)
Tocilizumab (Actemra®)	IL-6Rα	Intracardiac inoculation of MDA-MB-231 *in vivo*; metastatic trastuzumab-resistant SUM-159-HER2+-PTEN^-^ cells implanted into MFP mouse model to analyze tocilizumab +/- perifosine compared to docetaxel, trastuzumab-resistant BT474-PTEN^-^ xenograft mouse model to assess tocilizumab +/- trastuzumab; MFP xenograft mouse model injected with MCF10A-Erb2*, MDA-MB-361, BT-474, or HCC1954 cells and PDX xenograft mouse model; MDA-MB-231 and 4T1 mammosphere assays *in vitro*; Tamoxifen-resistant cell line, LCC2, used in xenograft mouse model and analyzed tocilizumab +/- tamoxifen *in vivo*.	(137) (78, 122, 123, 138)
Diacerein	IL-6Rα	MDA-MB-231 xenograft mouse model.	(139, 140)
Manuka Honey	IL-6Rα	*In vitro* findings using MDA-MB-231 cells.	(141)
Tubulosine	IL-6Rα/gp130	*In vitro* findings using MCF10A, Hs578T, MCF-7, MDA-MB-231, and MDA-MB-468 cells.	(142)
Chikusetsusaponin IVa Butyl Ester (CS-IVa-Be)	IL-6Rα	*In vitro* findings using MCF-7 and MDA-MB-231 cells.	(143)
Bazedoxifene	gp130	Xenograft mouse model inoculated with SUM159 or MDA-MB-231 cells in MFP and both sides of flank area.	(144, 145)
Raloxifene	gp130	*In vitro* findings using SUM-159 cells; *in vitro* findings using MDA-MB-231 cells.	(144, 146)
Ruxolitinib	JAK1/2	Treated as a single agent in MFP xenograft mouse model injected with MCF10A-Erb2*, HCC-70, T47D, or MDA-MB-231 cells, treated +/- trastuzumab in MFP mouse model inoculated with either PDX or MDA-MB-361, BT-474, or HCC1954 cells, transgenic MMTV-ErB2 +/- trastuzumab.	(138)
Glyceryl Trinitrate	JAK2	*In vivo* findings using 4T1 cells inoculated in right flank using syngeneic mouse model.	(147)
Pentadecanoic acid	JAK2	*In vitro* findings using normal MCF10A and MCF-7 stem-like cells (MCF-7/SC).	(148)
1-ferrocenyl-3-(4-methylsulfonylphenyl)propen-1-one	JAK2	*In vitro* findings using MCF-7 cells.	(149)
LYF-11	JAK2	*In vitro* findings using MCF-7 cells.	(150)
Withaferin A	JAK2	*In vitro* findings using MCF-7 and MDA-MB-231 cells.	(151)
AG490	JAK2	*In vitro* findings using MDA-MB-231 cells.	(152)
Naphtho[1,2-b]furan-4,5-dione (NFD)	JAK2	*In vitro* findings using MDA-MB-231 cells.	(153)
3-deoxy-2β,16-dihydroxynagilactone E	JAK2	*In vitro* findings using MDA-MB-231, MDA-MB-453, MDA-MB-468, and A549 cells.	(154)
Tagalide A	JAK2	*In vitro* findings using MDA-MB-231, MDA-MB-453, SKBR3, MCF-7, MT-1, ZR-75-1 cells.	(155)
Ganoderic acid A	JAK2	*In vitro* findings using MDA-MB-231 cells.	(156)
Methylseleninic acid	JAK2	*In vitro* findings using 4T1 cells, and use of syngeneic MFP mouse model using 4T1 cells.	(157)
7β-(3-Ethyl-cis-crotonoyloxy)-1α-(2-methylbutyryloxy)-3,14-dehydro-Z-notonipetranone (ECN)	JAK1/2	*In vitro* findings using MDA-MB-231 cells. *In vivo* findings using MFP xenograft mouse model using MDA-MB-231 cells.	(155)
Stattic	STAT3	Identification of Stattic and *in vitro* findings in MDA-MB-435S and MDA-MB-456 cells; Stattic treatment decreases cell survival of MCF7-HER2 cells *in vitro*; *In vitro* findings with doxorubicin on ZR-75-1 breast cancer cells.	(113, 158, 159)
STA-21	STAT3	*In vitro* findings using MDA-MB-231, MDA-MB-435s, and MDA-MB-468 cells *in vitro.*	(160)
FLLL31/FLLL32	STAT3	Xenograft mouse model inoculated with MDA-MB-231 cells in flank.	(161)
6a	STAT3	*In vitro* findings using MDA-MB-231 cells.	(162)
LLL12	STAT3	Inoculated MDA-MB-231 cells in right flank in xenograft tumor mouse model.	(163)
CDDO-Me	STAT3	*In vitro* findings using MDA-MB-468 cells; *in vivo* findings of CDDO-Me and its impact on breast tumor microenvironment.	(164, 165)
Naringenin	STAT3	*In vitro* findings using MDA-MB-231 cells.	(166)
Ilamycin C	STAT3	*In vitro* findings using MDA-MB-231, BT-549, MCF-7, and normal MCF10A cells.	(167)
Esculentoside A	STAT3	Reduces IL-6/STAT3 signaling through targeting breast CSCs, and inoculated murine breast CSCs (EMT6M) in syngeneic xenograft mouse model.	(168)
Catechol	STAT3	*In vitro* findings using MDA-MB-231 and MCF-7 cells.	(169)
Dihydrotanshinone	STAT3	*In vitro* findings using MCF-7 and MDA-MB-231 cells. MFP xenograft mouse model inoculated with MCF7 cells.	(170)
WP1066	STAT3/JAK2	*In vitro* findings in MDA-MB-231BR and BT-474BR cells; WP1066 reduces brain metastasis incidence *in vivo*	(94)
DT-13	gp130/STAT3	*In vitro* findings using MDA-MB-231 and MDA-MB-468 cells.	(171)
S3I-201	STAT3	*In vitro* findings using MDA-MB-231, MDA-MB-435, and MDA-MB-468 cells, and suppresses tumor growth of MDA-MB-231 tumors *in vivo*.	(172)
Cucurbitacin E	STAT3/JAK2	*In vitro* findings in MDA-MB-231 and Bcap37 cells.	(173)
5,15-diphenylporphyrin	STAT3	Usage of MDA-MB-435 cells *in vitro.*	(174)
Sabutoclax	STAT3	Bcl-2 antagonist, sabutoclax, reduces MCF7/A02 CSC population through inhibiting IL-6/STAT3 signaling.	(175)
Niclosamide	STAT3	Usage of MDA-MB-468 and MCF-7 cells *in vitro.*	(176)
Galiellalactone and two analogues (SG-1709 and SG-1721)	STAT3/JAK1/2	*In vitro* findings in MDA-MB-468 cells; analysis of combination with radiotherapy; *in vivo* treatment using breast xenograft tumor growth *in vivo*.	(177)
Nifuroxazide	STAT3	*In vitro* findings using MCF-7, MDA-MB-231, and 4T1 cells *in vitro*; Nifuroxazide suppressed *in vivo* investigation using 4T1 mouse model and analysis of lung metastases *in vivo.*	(178)
LLY17	STAT3	Usage of T47D cells *in vitro*, MDA-MB-468, MDA-MB-231, SUM159, and BT-549 cells *in vitro.*	(179)
Schisandrin A	STAT3	*In vitro* findings using MCF7 cells.	(180)
6Br-6a	STAT3	*In vitro* findings in MDA-MB-231 and MCF-7 cells; MDA-MB-231 mouse xenograft tumors *in vivo*	(181)
Pyrimethamine	STAT3	*In vitro* findings using TUBO and TM40D-MB.	(182)
Pectolinarigenin	STAT3	Findings using MCF-7, 4T1, and MDA-MB-231 cells *in vitro;* 4T1 breast cancer lung metastasis mouse model *in vivo*	(183)
Flubendazole	STAT3	*In vitro* findings using MDA-MB-231, Hs578T, BT-549, and 4T1 cells. *In vivo* metastasis models using 4T1-derived stem-like cells.	(184)
Eupalinolide J	STAT3	*In vitro* findings using HEK293 and MDA-MB-468 cells.	(185)
Betulinic acid	STAT3	*In vitro* findings using 4T1 and MDA-MB-231 cells. *In vivo* syngeneic subcutaneous mouse model using 4T1 cells.	(186)
Napabucasin	STAT3	*In vitro* findings using MDA-MB-231 cells.	(187)
Coumarin-benzo[b]thiophene 1, 1-dioxide	STAT3	*In vitro* findings using MDA-MB-231, HCT-116, MCF-7, and MCF-10 A cells. *In vivo* subcutaneous mouse model using 4T1 cells.	(188)
Carfilzomib	STAT3	*In vitro* findings using MDA-MB-231 cells.	(189)
Deguelin	STAT3	Usage of MDA-MB-231, MDA-MB-468, BT-549, and BT-20 cells *in vitro*; Deguelin reduced tumor growth of MDA-MB-231 cells *in vivo.*	(190)
Picrasidine G	STAT3	MDA-MB-468 cells compared to other breast cancer cells *in vitro*; Picrasidine G increases apoptosis in MDA-MB-468 cells *in vitro.*	(191)
Cantharidin	STAT3	*In vitro* findings in MDA-MB-231 cells.	(192)

**Table 2:** Clinical Studies Targeting IL-6/JAK/STAT3 in Breast Cancer.

Compound	Target	Brief Summary	Citation
Tocilizumab	IL-6Rα	Tested in combination with trastuzumab and pertuzumab in metastatic trastuzumab-resistant HER2+ breast cancer patients (Phase I: Completed); Under current investigation for treatment of COVID-19 in breast cancer versus non-cancer patients (Phase II); Immunotherapy-based treatment combinations in metastatic or inoperable locally advanced TNBC under current investigation (Phase Ib/II)	NCT03135171, NCT04871854, NCT03424005
Sarilumab	IL-6Rα	Combination therapy with capecitabine in metastatic TNBC (Phase I), and in Stage I-III TNBC with high risk residual disease (Phase II).	NCT04333706
Ruxolitinib	JAK1/2	Combined with capecitabine in advanced or metastatic HER2- breast cancer (Phase II: Terminated); Investigated in pSTAT3+ patients with metastatic or unresectable locally advance breast cancer (Phase II: Terminated); Combination therapy with Trastuzumab in metastatic HER2+ breast cancer (Phase I/II: Completed); Evaluated combination therapy with paclitaxel in advanced or metastatic breast cancer (Phase I/II: Completed); Combination therapy with exemestane in ER+ advanced breast cancer (Phase II: Completed); Current investigation of combination therapy with paclitaxel, doxorubicin, or cyclophosphamide in TNBC (Phase II); Under current investigation of combination therapy with pembrolizumab in metastatic stage IV TNBC (Phase I); Under current clinical investigation in patients with high risk and premalignant breast conditions (Phase II).	NCT02120417; NCT01562873; NCT02066532; NCT02041429; NCT01594216; NCT02876302; NCT03012230; NCT02928978 (193–195)
TTI-101	STAT3	TTI-101 given by mouth; administration of TTI-101 in mice blocked growth of multiple cancers including breast and was safe at high doses; Phase I study of TTI-101 in patients with advanced cancers as an interventional clinical trial.	NCT03195699

## Current Therapeutic Applications of IL-6 Signaling in Breast Cancer

Due to the heterogeneity of breast cancer, molecular classifications help determine which tumors may respond to targeted therapy. Each breast cancer subtype corresponds to a different prognosis and treatment regimen. Patients with luminal A and B breast cancer subtypes typically respond to targeted treatments such as tamoxifen, fulvestrant, or aromatase inhibitors; luminal patients have the most treatment options and better prognoses (196). HER2-enriched breast cancers initially respond to anti-HER2 antibodies such as Food and Drug Administration (FDA)-approved traustuzumab, lapatinib, pertuzumab, ado-trastuzumab emtansinse, and fam-trastuzumab (197–200), while TNBC patients have limited to no treatment options. Since TNBC tumors lack expression of ER, PR, and HER2, TNBC patients lack sensitivity to endocrine and molecular targeted treatments. Current SOC for TNBC patients includes systemic neoadjuvant chemotherapy and surgical resection (201). HER2-enriched breast cancer and TNBC are considered to be the most aggressive subtypes and maintain a higher propensity to metastasize (202). Metastatic HER2-enriched patients commonly acquire resistance to HER2-targeted therapies within one year, emphasizing the importance in developing novel therapeutics to treat or sensitize metastatic HER2-enriched tumors to current SOC treatment (203). Current findings elucidating the role of IL-6 in breast cancer progression, metastasis, and anti-cancer immunity, suggest the IL-6/JAK/STAT3 signaling pathway is an actionable target with preclinical and clinical studies demonstrating therapeutic potential in both primary and metastatic breast cancer. Notably, inhibiting the IL-6/JAK/STAT3 signaling axis has been investigated through directly targeting either IL-6, IL-6Rα, gp130 receptor, JAKs, or STAT3.

### IL-6 Inhibitors

The FDA has yet to approve IL-6/JAK/STAT3 pathway inhibitors for breast cancer. However, monoclonal antibodies (mAb) and small molecule inhibitors are under preclinical (**Table 1**) and clinical (**Table 2**) investigation. Siltuximab is a chimeric IL-6 mAb which received FDA-approval for the treatment of multicentric Castlemans disease in 2014 (204). Morancho and others examined the efficacy of siltuximab in several PDX models, and found only two of the six lines responded to siltuximab treatment. Contradictory to previous findings, they did not find a significant reduction in pSTAT3 in all PDX cultures after inhibiting IL-6, indicating that identification of IL-6-dependent tumors is important for anti-IL-6 therapies to be efficacious (134). For example, serum IL-6 may be used as a biomarker for IL-6-mediated treatment. Casneuf et al. analyzed the IL-6 serum levels of ERα-positive breast cancer patients and found IL-6 sera levels to be significantly correlated with intratumoral pSTAT3 protein expression. Furthermore, pretreatment of siltuximab reduced tumor growth in an ERα-positive breast cancer xenograft mouse model. Casneuf et al. also investigated a combination treatment using siltuximab and fulvestrant, and found combination treatment to attenuate tumor growth suggesting that IL-6/JAK/STAT3 combination therapy may sensitize tumors to SOC treatment (135). MEDI5116 is a novel anti-IL-6 mAb which neutralizes IL-6, is efficacious against HER2+ trastuzumab-resistant tumors, suppresses NF-κB signaling, and lung metastases (136). NF-κB promotes an IL-6 feed-forward inflammatory loop, whereas interruption of IL-6/NF-κB signaling may counteract IL-6-induced breast cancer chemoresistance and requires further investigation (102).

### IL-6Rα Inhibitors

Multiple anti-rheumatic agents targeting IL-6, IL-6Rα, and JAKs have gained FDA-approval and have transformed treatment outcomes for autoimmune and inflammatory diseases. In 2010, tocilizumab was the first approved in the United States for the treatment of rheumatoid arthritis (RA). Tocilizumab is a humanized anti-IL-6Rα mAb that competitively binds IL-6Rα and disrupts the IL-6/IL-6Rα complex in both classic and trans-signaling. Tociluzumab has a favorable safety and toxicity profile, and is now used for the treatment of juvenile idiopathic arthritis, adult-onset still’s disease, giant cell arthritis, chimeric antigen receptor T cell-induced cytokine release syndrome, and systemic associated-interstitial lung disease (205–207). In 2021, Tocilizumab received an emergency use authorization for the COVID-19 patients above the age of 2 years old (208).

Due to its diverse application, tocilizumab has been investigated in multiple cancers extensively, including breast cancer. Direct inhibition of IL-6Rα using tocilizumab effectively sensitizes resistant ER+ cells to tamoxifen *in vitro* and *in vivo* (78). HER2+ cells treated with tocilizumab or ruxolitinib, a JAK1/2 inhibitor, had reduced pSTAT3 protein expression, and increased cell apoptosis. Tocilizumab suppresses tumor volume, pSTAT3 protein expression, and cell proliferation (Ki67) in HER2+ orthotopic xenograft tumors (138). Administration of tocilizumab also reduces IL-6-mediated tumor growth, breast CSCs, and the development of secondary metastases in a PTEN^-^/HER2+/trastuzumab-resistant xenograft mouse model (122). Furthermore, tocilizumab inhibits TNBC mammosphere formation and suppresses mRNA expression of stemness markers: CD44, CD133, ALDH-1, EpCAM, Snail, Nanog, Oct-4, and Sox2 (123). Utilizing a TNBC intracardiac mouse model to model metastases, tocilizumab significantly suppresses bone metastases and osteoclast formation *in vivo* (137). Additionally, Jin et al. reported TNBC cells to secrete IL-6 in order to communicate with lymphatic endothelial cells to produce chemokine (C-C motif) ligand 5 (CCL5) to upregulate breast cancer lymph node metastasis. Combination treatment of tocilizumab and maraviroc, a CCR5 inhibitor, significantly reduces migratory and invasive phenotypes in TNBC cells *in vitro*, and breast cancer metastases in a TNBC xenograft mouse model *in vivo* (209). Given these results, tocilizumab proceeded to multiple clinical investigations (**Table 2**).

Tocilizumab was recently investigated in combination with trastuzumab and pertuzumab in metastatic trastuzumab-resistant HER2+ breast cancer patients in a Phase I clinical trial. This clinical study was completed March 20, 2020 and results are still under review (NCT03135171). Interestingly, tocilizumab is also under clinical investigation for severe COVID-19 treatment in breast cancer versus non-cancer patients where SOC chemotherapy may exacerbate severity of COVID-19 infection (NCT04871854). Tocilizumab in combination with atezolizumab and nab-paclitaxel is also under clinical investigation for safety of immunotherapy-based combination treatment in metastatic or inoperable locally advanced TNBC (NCT03424005). Sarilumab, an additional FDA-approved anti-IL-6Rα mAb for RA, which blocks both membrane-bound and soluble IL-6Rα, is under current Phase I and II clinical investigation in combination with capecitabine in stage I-III TNBC and metastatic TNBC patients (NCT04333706).

While IL-6Rα mAbs, tocilizumab and sarilumab, have made recent headway in clinical studies for breast cancer, drug repurposing remains an attractive therapeutic strategy to minimize the expensive, time-consuming drug development process (210). Diacerin, a non-steroidal anti-inflammatory drug used to treat osteoarthritis, directly interacts with IL-6Rα to suppress IL-6-induced phosphorylation of gp130, JAK1/2, STAT3, and MAPK in two TNBC cell lines. Furthermore, diacerin inhibits IL-6-induced STAT3 nuclear localization and transcriptional activity in TNBC cells, and significantly reduces tumor volume and induces apoptosis when compared to vehicle treated mice. Diacerin treatment reduces protein expression of IL-6Rα, pSTAT3, pMAPK, pAKT in TNBC tumor sections indicating diacerin could inhibit multiple IL-6-regulated oncogenic pathways (139).

Another strategy uses natural products as anti-cancer therapies. Aryappalli and colleagues report Manuka honey antagonizes IL-6Rα which inhibits downstream gp130, pJAK2, and pSTAT3; Manuka honey flavonoids, luteolin, chrysin, quercetin, and galangin disrupt IL-6 binding to IL-6Rα (141). Investigation of anti-cancer mechanisms of tubulosine, originally isolated from bark of *Pogonopus tubulosus* in 1964, identified tubulosine as a potent inhibitor of JAK2/STAT3 signaling through disruption of IL-6/IL-6Rα/gp130 complex formation (142). Furthermore, a triterpenoid saponin extracted from traditional Chinese medicine, Chikusetsusaponin IVa Butyl Ester (CS-IVa-Be), exhibits immunomodulatory effects by directly binding and antagonizing IL-6Rα. CS-Iva-Be reduces IL-6-induced STAT3 transactivation, TNBC cell viability, and synergizes with TRAIL to induce apoptosis in MDA-MB-231 cells (143). Overall, modulating IL-6/IL-6Rα interaction shows promising results in all subtypes of breast cancer mediated by IL-6/JAK/STAT3 signaling.

### gp130 Inhibitors

The gp130 receptor has evolved as an attractive therapeutic target to prevent downstream IL-6 signaling. Interestingly, small molecules which are FDA-approved for other therapeutic implications have been identified to have gp130 inhibitory effects. Since gp130 is the signal-transducing subunit for all IL-6 family cytokines, few gp130 inhibitors are able to maintain selectivity against IL-6. Bazedoxifene, an FDA-approved selective estrogen receptor modulator (SERM) with conjugated estrogens, was previously identified to reduce breast cancer cell proliferation and downregulate ERα and cyclin D1; however, its antitumor mechanism was not elucidated until recently (211). Interestingly, since the IL-6 family of cytokines bind different regions on the surface of gp130, bazedoxifene is able to selectively inhibit IL-6-induced STAT3 in TNBC both *in vitro* and *in vivo* through direct binding of the gp130’s D1 domain (144, 145). Bazedoxifene was identified using a multiple-ligand simultaneous docking and drug repositioning approach in order to identify a small molecule that was able to directly bind into “hot-spot” residues on gp130 to prevent protein-protein interactions between IL-6 and gp130. Bazedoxifene inhibits STAT3-mediated transcriptional activity and, in turn, suppresses breast cancer colony formation, migration, and invasion. Bazedoxifene also reduces TNBC tumor volume suggesting the translational potential of the compound as an IL-6/JAK/STAT3 inhibitor (145). Whether bazedoxifene can inhibit IL-6-induced metastatic formation in TNBC is not known. Since bazedoxifene is FDA-approved with a favorable safety profile, bazedoxifene may provide clinical utility as a repurposed compound for the treatment of TNBC, but requires further investigation. Raloxifene, an additional FDA-approved SERM, has also been identified to directly bind to gp130 and suppress STAT3 activation in a TNBC cell line, SUM-159 (144). To further complement these findings, another group identified raloxifene to suppress breast cancer cell viability using another TNBC cell line, MDA-MB-231 (146). In 2007, raloxifene gained FDA-approval for the prevention of invasive breast cancer in postmenopausal women (212, 213). Currently, raloxifene is not approved for treatment of breast cancer. Of note, bazedoxifene and raloxifene are both FDA-approved for the prevention of postmenopausal osteoporosis, and have been reported to prevent bone loss and increase bone mineral density (214). Since the bone is a common distant site of metastasis in breast cancer, and breast cancer bone metastatic patients suffer from microfractures and severe pain, raloxifene and bazedoxifene may provide additional benefits in addition to treating primary breast tumors (215).

### JAK Inhibitors

Another approach to targeting the IL-6/JAK/STAT3 signaling axis is through direct JAK inhibition of one or multiple JAK family of enzymes. Tofacitinib, ruxolitinib, baricitinib, and upadacitinib are all FDA-approved JAK inhibitors for implications other than breast cancer, e.g. RA, psoriatic arthritis, severe ulcerative colitis, polyarticular course juvenile idiopathic arthritis, myelofibrosis (216–220). Ruxolitinib is a bioavailable tyrosine kinase inhibitor of both JAK1 and JAK2, and was investigated in metastatic TNBC patients (NCT01562873). pSTAT3-positive metastatic TNBC patients were enrolled in a non-randomized Phase II study to examine ruxolitinib safety and efficacy. Although ruxolitinib was well-tolerated and exhibited on-target activity, this clinical study did not reach its primary efficacy endpoint indicating alternative mechanisms may mediate resistance (193). To potentially overcome this barrier, ruxolitinib is under current clinical investigations to examine combination treatments with paclitaxel, doxorubicin, cyclophosphamide, or pembrolizumab in TNBC patients (**Table 2**) (NCT03012230; NCT02928978). Furthermore, additional JAK inhibitors have been investigated preclinically and are demonstrated to be efficacious *in vivo*. Glyceryl trinitrate inhibits JAK2 through s-nitrosylation to suppress IL-6-induced migration and invasion in TNBC cells. Additionally, glyceryl trinitrate infusion decreases lung metastatic lesions in a TNBC syngeneic mouse model (147). Pentadecanoic acid suppresses the CSC subpopulation through inhibition of IL-6/JAK/STAT3 signaling and increases apoptosis in ER+ breast cancer cell line; however, the exact mechanism remains unknown (148). *In vitro* evidence identified a ferrocene derivative, 1- ferrocenyl-3-(4-methylsulfonylphenyl)propen-1-one (FMSP), that reduces IL-6-induced downstream effectors, CSC renewal, and downregulates stemness markers: Wnt1, Notch1, β-catenin, SOX2, CXCR4, and ALDH1A1 (149). Liu et al. investigated multiple derivatives of 2-phenyl-1,8-naphthyridin-4-one and identified LYF-11 which blocked IL-6-mediated EMT through the suppression of phosphorylated JAK2 (150). Direct mechanisms and efficacy *in vivo* of inhibitors listed above remain to be investigated. Other JAK inhibitors investigated preclinically in breast cancer include withaferin A, AG490, naphtho[1,2-b]furan-4,5-dione, 3-deoxy-2β,16-dihydroxynagilactone E, tagalide A, ganoderic acid A, methylseleninic acid, and 7β-(3-Ethyl-cis-crotonoyloxy)-1α-(2-methylbutyryloxy)-3,14-dehydro-Z-notonipetranone (**Table 1**). While JAK inhibition remains heavily studied in multiple cancers, the FDA has administered safety warnings against JAK inhibitors underscoring the need to investigate additional approaches to target the IL-6/JAK/STAT3 pathway (221).

### STAT3 Inhibitors

STAT3 has gained significant attraction as an actionable anti-cancer therapeutic; however, there are currently no FDA-approved STAT3-targeted therapies for the treatment of cancer. Therefore, multiple studies have investigated novel small-molecule compounds which negatively regulate STAT3 activation in breast cancer (222) (**Table 1**). Strategies for STAT3 inhibition include disruption of STAT3 phosphorylation, dimerization, nuclear translocation, or prevention of DNA binding. In 2006, stattic was identified as a small-molecule that disrupts the src homology-2 (SH2) domain of STAT3, and thereby prevents STAT3 recruitment to gp130 on the cell membrane (158). Functionally, increasing doses of stattic was able to prevent STAT3 dimerization and nuclear translocation resulting in a subsequent decrease in IL-6-induced pSTAT3. Stattic induces apoptosis in TNBC cells (158). Since its discovery, others have identified stattic to be efficacious against breast CSCs through the downregulation of STAT3 stemness genes Oct-4, Sox-2, and Slug (113). Combination studies also reveal stattic is synergistic with SOC therapeutic, doxorubicin, and suppresses anti-apoptotic genes, Bcl-2 and Bcl-xL, to promote breast cancer cell apoptosis (159). Interestingly, another STAT3 inhibitor, STA-21, also directly binds to the SH2 domain of STAT3 to repress STAT3 transcriptional activity, and is efficacious in TNBC cells *in vitro* (160). Utilizing a structural-based computational screening approach, S3I-201 was identified to target the SH2 domain of STAT3 and suppress downstream signaling to induce breast cancer cell apoptosis and exhibit activity *in vivo* in a TNBC mouse model (172). Additionally, small molecule STAT3 inhibitors, FLLL31 and FLLL32, are derivatives of curcumin and selectively bind to the JAK2 and STAT3 SH2 domain. The JAK2 and STAT3 SH2 domain is essential for STAT3 phosphorylation, therefore, inhibition disrupts STAT3 dimerization and translocation required for activation of STAT3 transcriptional activity. Subsequently, downstream STAT3 target genes are significantly downregulated upon increasing doses of FLLL31 and FLLL32. FLLL31 exhibits efficacy *in vivo* where systemic administration reduced tumor growth and vascularity in a TNBC xenograft mouse model (161).

Other novel molecules have been identified to exhibit anti-STAT3 activity by inhibiting STAT3 phosphorylation. LLL12, prevents IL-6-induced STAT3 phosphorylation at Y705, and demonstrates efficacy *in vivo* in a TNBC MFP mouse model with a concomitant reduction in tumor volume and pSTAT3 expression (163). Interestingly, novel JAK2/STAT3 inhibitor, WP1066, can penetrate the blood-brain-barrier, suppress brain metastases *in vivo*, and prolong overall survival in mice inoculated with brain-trophic TNBC breast cancer cells *via* an intracardiac injection. WP1066 also reduces breast cancer cell viability and cell invasion in brain-trophic breast cancer cells through the reduction of STAT3 target genes, MMP-9 and VEGFR2 (94). Furthermore, Zinzalla and colleagues synthesized multiple pyrrolidinesulphonylaryl molecules and identified compound, 6a, to selectively inhibit IL-6-induced pSTAT3 in TNBC cells, and inhibit cell growth in STAT3-dependent but not STAT3-null cells demonstrating its dependency on STAT3 for inhibition (162). Additional novel STAT3 inhibitors that have been investigated preclinically for the treatment of breast cancer include: LLY17, 6Br-6a, napabucasin, and coumarin-benzo[b]thiophene 1, 1-dioxide conjugates (**Table 1**) (179, 181, 187, 188).

Interestingly, multiple natural compounds have been identified to suppress STAT3 activity and exhibit anti-cancer properties. For example, CDDO-Me is a triterpenoid with anti-inflammatory properties and suppresses activated STAT3 protein expression, nuclear translocation, and STAT3 anti-apoptotic genes in ovarian and breast cancer *in vitro* (164). Other natural compounds which have exhibited anti-cancer properties in breast cancer through modulation of STAT3 activity include: naringenin, ilamycin C, esculentoside A, catechol, dihydrotanshinone, DT-13, cucurbitacin E, galiellalactone, schisandrin A, pectolinarigenin, eupalinolide J, betulinic acid, deguelin, picrasidine G, and cantharidin (**Table 1**) (166–171, 173, 177, 180, 183, 185, 186, 190–192). Compounds have also been repositioned for the treatment of breast cancer due to their anti-cancer activity through inhibition of STAT3, and are under preclinical investigation. For example, niclosamide is currently FDA-approved as an anti-parasitic drug, yet treatment exhibited inhibition of IL-6-induced STAT3 activation resulting in suppression of adipocyte-induced EMT in breast cancer cells (176). Additionally, nifuroxazide, an antibiotic, exhibits anti-STAT3 activity and suppresses breast cancer tumor growth and lung metastases (178). Additional repurposed compounds under preclinical investigation for inhibition of STAT3 activity include pyrimethamine, flubendazole, and carfilzomib (**Table 1**) (182, 184, 189). While STAT3 inhibitors have been extensively investigated preclinically, only one compound is under current clinical investigation. TTI-101 is a novel small molecule STAT3 inhibitor, and is in a Phase I clinical trial examining pharmacokinetics and compound safety in advanced breast cancer patients as well as patients with unresectable solid tumors (NCT03195699).

## Conclusions

Under normal conditions, IL-6 is an important regulator in acute phase immune responses and modulates both anti- and pro-inflammatory reactions. Breast cancer cells can hijack the IL-6/JAK/STAT3 signaling to evade normal immune responses and further promote tumor growth by activating surrounding microenvironmental cells. Therefore, it remains pertinent to maintain a homeostatic balance of IL-6/JAK/STAT3 as dysregulation creates a vicious autocrine and paracrine inflammatory loop which promotes breast cancer metastasis and therapeutic resistance. Recent reports extensively elaborate on IL-6’s pleiotropic effects and pro-metastatic role in breast cancer; however, current evidence on whether IL-6 promotes site-specific metastases requires further investigation. Due to recent evidence of IL-6 inducing the CSC subpopulation and mediating therapeutic resistance in breast cancer, preclinical investigations in metastatic breast cancer focus on targeting this pathway with either mAbs, novel small molecule compounds, or by repurposing current FDA-approved compounds. Multiple actionable therapeutic targets reside in the IL-6 pathway including inhibition of IL-6 directly, IL-6Rα, gp130 receptor, JAKs, or STAT3. While there remains a plethora of preclinical studies analyzing IL-6/JAK/STAT3 inhibitors on breast cancer growth, there remains an urgent gap analyzing compound efficacy against breast cancer metastases *in vivo.* Additionally, since IL-6 modulates multiple physiological processes and oncogenic pathways, elucidating effective biomarkers for breast cancer patients who could benefit from targeted IL-6/JAK/STAT3 inhibitors could aid in the development of therapeutics for metastatic breast cancer patients.

## Author Contributions

Designed the manuscript, SM. Selected the reviewed literature, SM. Compiled the review tables and figures, SM. Wrote the manuscript, SM. Contributed literature search, DD. Table preparation, DD, GW, and SM. Manuscript writing and editing, DD and GW. Supervised, H-WL. Helped design figures, H-WL. Edited, H-WL. Funded this publication, H-WL. All authors have read and agreed to the published version of the manuscript.

## Funding

The authors acknowledge funding support by NIH grants P30CA012197 (BP), 1R01CA228137-01A1 (H-WL), as well as, DoD grants, W81XWH-17-1-0044 (H-WL), W81XWH-19-1-0072 (H-WL), W81XWH-19-1-0753 (H-WL), W81XWH-20-1-0044 (H-WL) and F31CA261027-01A1 (DLD).

## Conflict of Interest

The authors declare that the research was conducted in the absence of any commercial or financial relationships that could be construed as a potential conflict of interest.

## Publisher’s Note

All claims expressed in this article are solely those of the authors and do not necessarily represent those of their affiliated organizations, or those of the publisher, the editors and the reviewers. Any product that may be evaluated in this article, or claim that may be made by its manufacturer, is not guaranteed or endorsed by the publisher.
